# Speed of processing training to improve cognition in moderate to severe TBI: a randomized clinical trial

**DOI:** 10.3389/fneur.2024.1445560

**Published:** 2024-08-29

**Authors:** Nancy D. Chiaravalloti, Silvana L Costa, Caroline Armknecht, Kristin Costanza, Sean Wallace, Nancy B. Moore, John DeLuca

**Affiliations:** ^1^Kessler Foundation, Center for Neuropsychology and Neuroscience Research, East Hanover, NJ, United States; ^2^Department of Physical Medicine and Rehabilitation, Rutgers –New Jersey Medical School, Newark, NJ, United States; ^3^Department of Neurology and Neurosciences, Rutgers –New Jersey Medical School, Newark, NJ, United States

**Keywords:** traumatic brain injury, episodic memory, processing speed, cognitive rehabilitation, speed of processing training, UFOV

## Abstract

**Background:**

Moderate to severe traumatic brain injury (TBI) often results in cognitive deficits. Processing speed (PS) deficits are common, exerting a significant impact on daily life. Few studies have examined the efficacy of cognitive rehabilitation specifically for PS deficits in moderate to severe TBI.

**Objective:**

Examine the efficacy of Speed of Processing Training (SOPT) in moderate to severe TBI. This protocol is a 10-session behavioral intervention for PS deficits that has been successfully used with other cognitively impaired populations.

**Methods:**

This double-blind, placebo-controlled, randomized clinical trial included 46 participants with moderate to severe TBI, 22 randomly assigned to the treatment group and 24 to the placebo-control group. Baseline and follow-up measures included a task similar to the training task (UFOV), measures of near transfer (neuropsychological measures of processing speed: Symbol Digit Modalities Test (SDMT), Wechsler Adult Intelligence Scale-IV (WAIS-IV) Symbol Search, WAIS-IV Coding) and measures of far transfer [neuropsychological measures of learning and memory: the California Verbal Learning Test-II (CVLT-II), Memory Assessment Scales - Prose Memory (MAS-PM)].

**Results:**

Significant improvement from pre-to post-SOPT was observed on all subtests of the UFOV, which is similar to the training task. There was no significant difference on neuropsychological measures of PS or new learning and memory post-treatment. Neuropsychological assessment 6-months post-treatment showed no significant change in PS ability over time. Monthly booster sessions did not impact performance at the 6-month follow-up.

**Conclusion:**

Consistent with the SOPT literature, SOPT improves PS ability as measured by the UFOV, a task similar to the training task, in moderate to severe TBI. However, neither near nor far transfer was noted. That is, no improvement was noted on neuropsychological measures of PS.

## Introduction

Traumatic Brain Injury (TBI) often leads to long-term medical complications including physical, emotional, and cognitive disabilities. Affected cognitive functions can include attention (1), processing speed (1), executive functions (2) and memory (1). Cognitive deficits can interfere with return to work, school, and family and social relationships (3). Processing Speed (PS) deficits are among the most common cognitive deficits post-TBI (4).

PS can be defined as either the amount of time it takes to process information, or the amount of information processed within a unit of time (5). PS is highly vulnerable to brain damage, with diminished PS noted in TBI, Multiple Sclerosis (MS), Parkinson’s disease, HIV, Chronic Fatigue Syndrome, dementia, and schizophrenia (6–10). Decreases in PS have also been shown to be a significant contributor to age-related decline in other cognitive domains (11). PS measures are highly sensitive and able to differentiate between clinical and healthy groups (12).

PS has been recognized as an important aspect of general cognition (13), well-documented as a component of intelligence and correlating with other abilities, such as verbal abilities (14), long term memory (15, 16), executive functions (17), visuospatial skills (18), and working memory (7, 16). Thus, an identified deficit in PS may impact other areas of cognition. Slowed PS can also result in real world problems, including safety concerns [e.g., driving (19)], difficulty with tasks of daily living (20) and occupational problems (21). Additionally, PS impairment is significantly correlated with decreased quality of life in clinical populations (22). Thus, deficits in PS have wide-reaching effects that could influence well-being. Consequently, improvements in PS are expected to result in improvements in everyday living and quality of life.

Following inpatient rehabilitation, individuals with TBI often continue with outpatient therapy to minimize physical and cognitive sequelae. The most frequent means by which cognitive deficits are treated is cognitive rehabilitation, demonstrated to be effective for ameliorating deficits in attention, working memory, visuospatial processing, and communication skills post TBI (23). An international expert panel convened to develop recommendations for managing cognitive deficits following TBI recommended the use of metacognitive strategies focused on daily life activities, failing to find sufficient evidence to support computer-based tasks targeting PS deficits in persons with TBI (24). The American Congress for Rehabilitation Cognitive Rehabilitation Task Force (CRTF) similarly recommended that treatment of deficits in attention/processing speed utilize metacognitive strategy training. However, the CRTF also advocated the incorporation of direct training techniques, noting that such training may improve aspects of attention with no evidence of negative effects (23). Yet, they clearly state there to be insufficient evidence to support benefits of such direct training compared with standard rehabilitation on functional outcomes. Thus, while direct training protocols for attention and PS deficits exist, evidence to support their application in TBI is insufficient (23, 24).

Speed of Processing Training (SOPT), a multi-session computer based behavioral intervention involving trainer-guided practice across 3-tasks (Target Detection, Discrimination and Localization) and progressively increasing task demands, has been shown to improve PS in several studies in healthy aging (20, 25). Gains made with SOPT have also been shown to generalize to the everyday environment (20, 25, 26). Taken together, these studies demonstrate SOPT to be an effective means of improving PS in the healthy aging population. Work from our group has recently shown SOPT to be an effective treatment for PS deficits in persons with MS with benefit observed on the training task (UFOV) as well as a neuropsychological task assessing PS (27). Less benefit was observed as neuropsychological outcome measures became more distinct in cognitive demands from SOPT, however some benefit was seen on daily life tasks (28). Long-term maintenance was observed.

The current study evaluated the efficacy of SOPT for improving PS in persons with moderate–severe TBI with documented PS deficits. We hypothesized that participants completing SOPT will exhibit improved performance on PS tests relative to the placebo control group and that participants completing SOPT will maintain a higher level of PS performance for 6 months following treatment relative to baseline. We additionally hypothesized that SOPT will lead to improvements on new learning and memory (NLM) relative to the control group, as past research has shown that processing speed deficits impact NLM (16, 29).

## Methods

### Participants

Fifty-six individuals with moderate–severe TBI were randomized to treatment condition. 46 individuals completed treatment and immediate follow-up (TX *n* = 22) or placebo control (pCTL *n* = 24). There were no significant differences between groups in age, injury severity, or estimated premorbid IQ (Table 1). The control group had a longer time since injury (ns, Hedges *g* = 0.6) and more years of education (ns, Hedges *g* = 0.47) than the treatment group. The treatment group had a higher proportion of males than pCTL (X^2^ = 8.85, *p* < 0.01). Inclusion criteria were: (1) PS impairment (At least one PS measure at least 1 standard deviation below the mean of normative data); (2) aged 18–70; (3) no neurologic history other than moderate to severe TBI; (4) no history of major depressive disorder, schizophrenia, or bipolar disorder; (5) no substance dependence/use disorder history; (6) Sufficient visual function to view stimuli (e.g., no scotomas; 20/60 minimum acuity); and (8) intact language comprehension. TBI severity was determined by review of medical records to meet at least one of four criteria for moderate to severe TBI (a) post-traumatic amnesia for more than 24 h; (b) trauma-related intracranial neuroimaging abnormalities; (c) loss of consciousness for more than 30 min, or (d) Glasgow Coma Scale score lower than 13, consistent with the NIDILRR TBI Model System criteria for moderate to severe TBI.

**Table 1:** Demographic and TBI characteristics by treatment group.

	Treatment *M* (SD) *n* = 22	Placebo control *M* (SD) *n* = 24	Test statistic *t*
Gender	87% male	46% male	**X**^**2**^ = 8.85*
Education	13.3 (1.8)	14.3 (2.4)	1.51
Age	40.39 (14.65)	42.5 (11.6)	0.55
Months since injury	88.1 (55.7)	171.1 (189.5)	1.52
Severity of TBI	9% moderate 91% severe	8% moderate 92% severe	X^2^ = 0.97
WASI vocabulary *t*-score (estimated pre- morbid verbal IQ)	43.96 (14.92)	41.58 (12.62)	−0.59
Verbal comprehension (Token test)	30.3 (2.1)	30.5 (2.7)	0.23

*p < 0.01.

### Study design

This 5-week, double-blind RCT employed a parallel groups design. Eighty (80) participants were randomized to group with a 1:1 allocation ratio (TX, pCTL) via a computerized random number generator prior to initiating data collection. Randomization and group assignment was done by the research manager, who did not have any other role in data collection or contact with the participants. Data collection ceased at conclusion of funding, at which time 56 participants were randomized. Treatment allocation was concealed via sealed envelopes. Each participant number received a group assignment via computerized random number generator, which were sealed in individual envelopes with the participant number printed on each. The research assistant who conducted the treatment was handed the sealed envelope just prior to session one and he/she was the only person to open the envelope and have knowledge of the randomization assignment. The individual responsible for group assignment was blind to assessment results and group assignment was verified by a second person via duplicate copy of the randomization.

Participants completed baseline testing following randomization, which included patient report of everyday cognition and neuropsychological assessment. The 10-session treatment then began. Within one-week of treatment completion, the immediate follow-up assessment (IFU) was completed, consisting of baseline procedures utilizing alternate forms. The treatment group was then randomized to a monthly booster or placebo-booster session group to examine the impact of booster sessions on maintenance of treatment gains over time. 6-months after the completion of treatment, assessment measures were again administered (long-term follow up: LTFU) for all participants, utilizing alternate forms when available.

Baseline, IFU, and LTFU assessments were completed by the same research assistant (RA), who were blind to group. Masking was preserved via several mechanisms: (1) Different RAs conducted treatment versus assessments (2) communication about participants occurred through the research coordinator, rather than between RAs. Participants were blind to group assignment, consenting to participate in a study examining the impact of cognitive/thinking computer-based exercises in which they had a 50/50 chance of being in the treatment group. Data were collected in a quiet testing room at a research facility.

Following a participant’s completion of the long-term follow-up evaluation, they completed a post-study questionnaire to assess blinding. 33% of participants identified group assignment correctly (less than chance); 25% of participants were incorrect and 41% said they had no idea.

There were no changes to the methodology once data collection was initiated.

### Power analysis

Analysis of covariance (ANCOVA) was the primary method for examining group differences. A non-directional test was planned with alpha set at 0.05. 25 subjects per group were needed to achieve power of 0.80. We documented an effect size of 1.35 in 49 TBI participants with the Letter Comparison (LC) PS test as the primary outcome. The SDMT (the primary outcome in the current study) correlated with LC at *r* = 0.853, *p* < 0.001 in this pilot sample. We thus targeted enrollment of 80 participants (40 per group) to allow for attrition in examining long term follow-up data.

### Treatment protocol

The treatment condition consisted of 10-SOPT sessions, occurring twice/week for 5 weeks; sessions lasted 45–60 min. SOPT includes computer-based exercises with trainer-guided practice, including Target Detection (target presence/absence, identification), Discrimination and Localization (same/different discriminations) (22, 30, 31). Several manipulations increased task demands. (1) Display speed from 17–500 ms. (2) Greater complexity and number of task demands (i.e., simultaneous auditory/visual task identification). (3) Peripheral task, central task, or both increasing in complexity. The trainer tailors the training task to the individual’s ability level. SOPT requires the participant to practice on many different stimuli at various stimulus durations, theoretically increasing generalization to daily life. A certification procedure is required for all trainers and a training manual is provided [see Ball et al. (32) for details].

Participants assigned to the pCTL condition met with the therapist for the same duration and frequency as the TX group. Sessions consisted of a computer-based training program in which they were taught various aspects of computer literacy across 10 sessions over 5 weeks. Sessions ranged in difficulty from the use of a mouse to using the internet to plan a vacation. The pCTL group was not exposed to SOPT.

### Outcome measures

Outcome was measured on a task similar to the training task, as well as at both near and far transfer. The UFOV, an assessment of visual PS administered via computer assessed change on a task similar to the training task, was administered to the treatment group only. Research has demonstrated the UFOV to predict functional outcomes, including vehicle crashes, in older adults; this was the primary outcome in a large study of SOPT in aging (ACTIVE). The UFOV consists of 3 subtests, speed, selective attention, and divided attention; all 3 subtests were utilized as dependent variables in this study.

Near transfer, the primary outcome, was assessed via several neuropsychological measures of PS [Symbol Digit Modalities Test (SDMT) (33), Wechsler Adult Intelligence Scale-IV (WAIS-IV) Symbol Search (34), WAIS-IV Coding (34) (DSMT)] at each assessment.

Far transfer, our secondary outcome, included a measure of NLM (California Verbal Learning Test-II, CVLT-II) (35), an objective measure of PS in daily life (Timed Instrumental Activities of Daily Living Test, TIADL (36), data presented elsewhere), as well as patient reports of quality of life [Traumatic Brain Injury – Quality of Life, TBI - QOL (37)], and emotional symptomatology (Beck Depression Inventory, BDI).

Neuropsychological testing characterized cognitive functioning, including tests of premorbid intelligence [WAIS-IV (34) Vocabulary], Attention (Digit Span), Working Memory (Letter Number Sequencing), Executive Functioning (Delis-Kaplan Executive Function System (38), Trail Making, Color-Word, Tower, Fluency), and NLM (39) (CVLT-II; Table 2).

**Table 2:** Neuropsychological functioning at baseline.

Variable	Treatment group *N* = 22	Control group *N* = 24	Test statistic *t*
**Attention**			
RBANS digit span scaled score	6.90 (3.82)	8.33 (3.92)	1.23
**Working memory**			
Letter number sequencing scaled score	8.0 (2.86)	7.42 (2.55)	−0.74
PASAT- trial 1 z score	−1.79 (1.03)	−1.91 (1.32)	−0.34
PASAT- trial 2 z score	−1.59 (0.93)	−1.90 (1.01)	−1.03
PASAT- trial 3 z score	−1.17 (0.68)	−1.45 (0.76)	−1.23
PASAT- trial 4 z score	−1.10 (0.63)	−1.30 (0.72)	−0.96
**Processing speed**			
SDMT z score	−2.57 (1.69)	−2.78 (2.11)	−0.29
WAIS-IV symbol search z score	−1.50 (0.85)	−1.51 (1.07)	−0.05
WAIS-IV Coding standard score	6.05 (3.29)	5.82 (3.46)	−0.22
**Executive functioning**			
Trail making, visual scanning scaled score	6.14 (3.95)	6.38 (4.94)	−0.17
Trail making number sequencing scaled score	6.67 (4.76)	5.42 (4.62)	−0.89
Trail making letter sequencing scaled score	6.10 (4.69)	5.04 (4.73)	−0.75
Trail making switching scaled score	5.71 (3.54)	4.58 (4.36)	−0.96
Verbal fluency letter scaled score	7.14 (3.15)	5.83 (3.79)	−0.017
Verbal fluency category scaled score	8.04 (3.88)	6.29 (4.59)	−1.41
Verbal fluency switching scaled score	7.30 (4.42)	6.04 (4.65)	−0.95
Color-word reading scaled score	4.87 (3.78)	5.04 (3.32)	0.17
Color word color naming scaled score	4.83 (3.81)	5.46 (3.62)	0.58
Color word inhibition scaled score	6.57 (4.43)	6.46 (4.48)	−0.08
Color word inhibition switching scaled score	5.13 (4.03)	5.04 (4.85)	−0.07
**Learning & memory**			
RBANS story memory immediate Z score	−1.50 (1.85)	−1.75 (1.42)	−0.5
RBANS story memory delayed Z score	−1.59 (1.91)	−2.17 (1.96)	−0.99
CVLT total learning T score	40.9 (14.2)	35.6 (16.7)	1.17
CVLT learning slope T1-5 z Score	−0.46 (1.47)	−0.96 (1.32)	1.23
BVMT-R total learning T Score	45.9 (17.6)	45.9 (11.7)	0.003
BVMT-R total recall T score	31.0 (15.0)	27.1 (10.9)	−1.00
BVMT-R delayed recall T score	34.1 (13.6)	28.7 (12.1)	−1.41

### Statistical analyses

All participants who had complete baseline to post-treatment data were included in all statistical analyses (TX = 22, pCTL = 24). Baseline performance was utilized as the covariate in paired sample *t*-tests (UFOV) and analysis of covariance. We hypothesized that the TX group would improve on all measures as compared with the pCTL. To examine long-term maintenance, we conducted a 2 (group: TX, pCTL) × 2 (follow-up: IFU, LTFU) RM-ANOVA on the SDMT, symbol search and coding.

SPSS version 20 (SPSS Inc., Chicago, IL) was used for data analysis. No interim analyses were performed. Standard protocol registrations, approvals and patient consents were obtained. All study procedures were approved by the Kessler Foundation Institutional Review Board. All participants provided written informed consent. No interim analyses were conducted. The clinical trial is registered with www.clinicaltrials.gov (protocol ID: NCT02020564).

## Results

**Figure 1:**
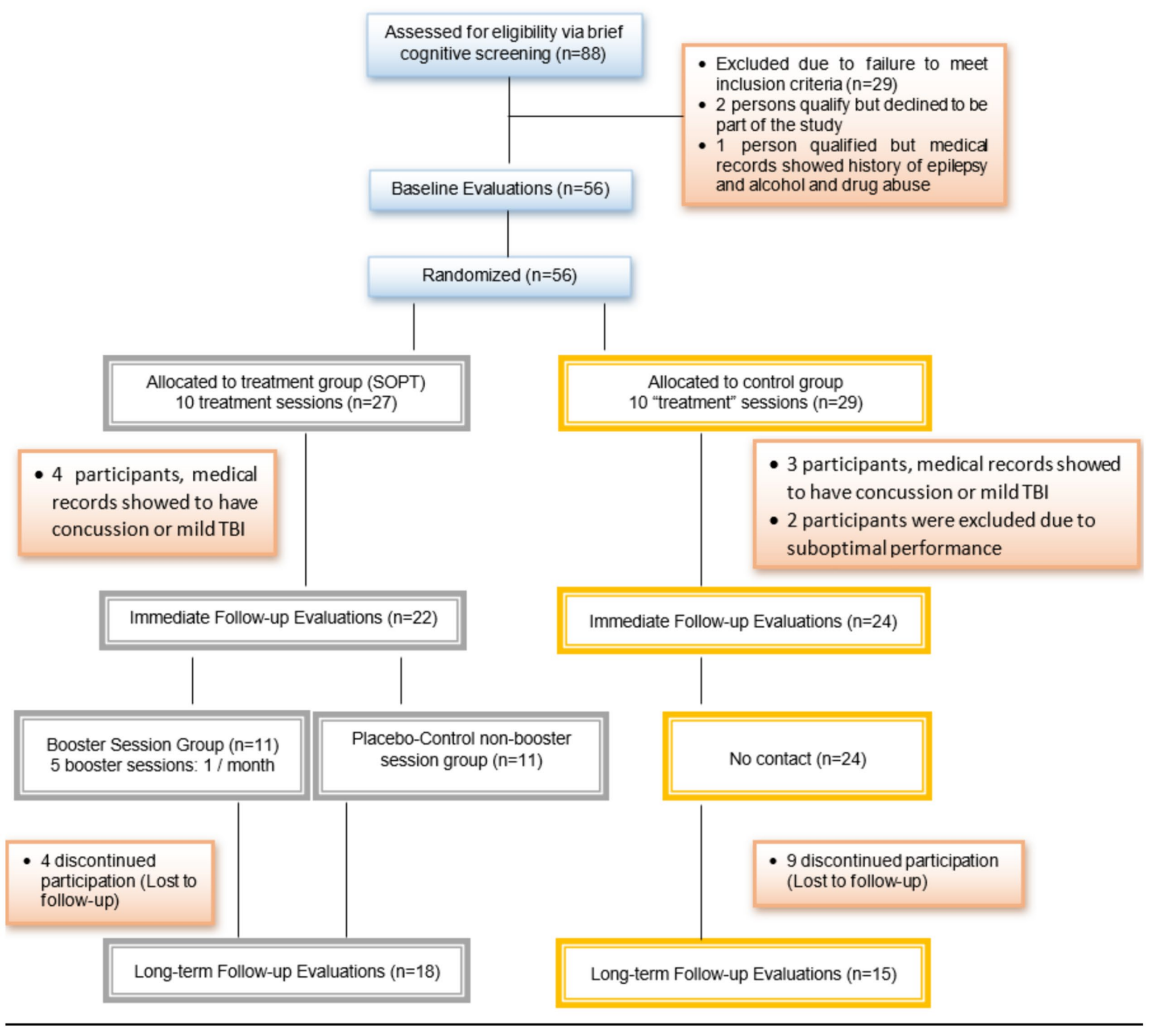
Consort flow chart.

Recruitment ran from 5/1/2013–4/1/2019. One enrolled participant dropped out prior to baseline due to loss of interest. One participant dropped out between baseline and IFU (2%). Thirteen (13) participants dropped out from IFU to LTFU (23%). One additional participant in the treatment group was excluded before LTFU due to a seizure. Additional reasons for dropout included participant relocation, health complications, and scheduling challenges. No related adverse events were noted. Analysis was based on intent-to treat population. In the case of missing data, the last observation was carried forward, conservatively assuming no change in the absence of data indicating otherwise (40) (see Figure 1 for the consort flow chart).

At baseline, there was no significant difference between the groups on any neuropsychological test administered (Table 2).

### Treatment efficacy

#### Neuropsychological performance

**Figure 2:**
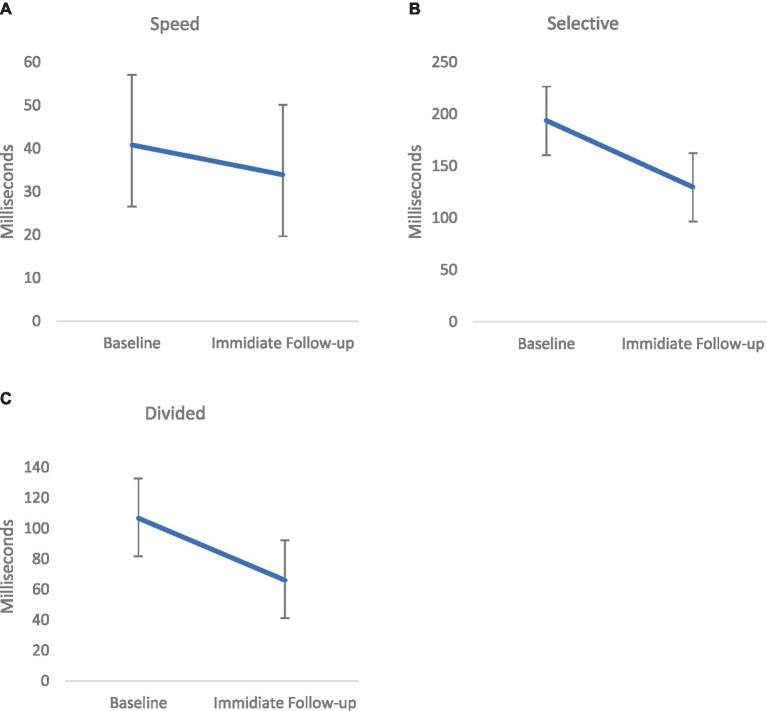
Mean performance on the 3 measures (Speed [*p* < 0.05; effect size=. Hedges *g* = 0.49, **(A)**], Selective Attention [*p* < 0.001, Hedges *g* = 0.47, medium effect, **(B)**], and Divided Attention [*p* < 0.01, Hedges *g* = 0.82, large effect, **(C)**]) of the Useful Field of View (UFOV) from Pre-to Post- treatment in the treatment group (performance is measured in speed; thus a lower score is better). Error bars represent Standard Error of the Mean (SEM).

Performance on the UFOV was evaluated pre and post treatment in the treatment group only to evaluate post-treatment changes on a task similar to the treatment task. Paired sample *t*-tests were conducted. A significant effect of training was noted on the UFOV speed [Task I; *t*(20) = 2.33, *p* = 0.03, Hedges *g* = 0. 49, medium effect; Baseline (*M* = 38.22, SD = 67.56) to IFU (*M* = 32.05, SD = 59.21)], Divided Attention [Task II; *t*(20) = 2.24, *p* = 0.037, Hedges *g* = 0.47, medium effect; Baseline (*M* = 102.28, SD = 109.57) to IFU (*M* = 65.35, SD = 104.11)] and Selective attention [Task III; *t*(20) = 3.92, *p* = 0.001, Hedges *g* = 0.82, large effect; Baseline (*M* = 182.45, SD = 139.95) to IFU (*M* = 123.07, SD = 139.92)]. Performance on the UFOV was significantly faster following treatment as compared with prior to treatment (Figure 2).

To evaluate near transfer of training related improvement, neuropsychological measures of PS were administered. No significant treatment effects were noted on any of the neuropsychological PS outcome measures, with small to medium effects sizes noted on the planned ANCOVAs (WAIS-IV Coding (partial *ƞ*^2^ = 0.039), Symbol Search (partial *ƞ*^2^ = 0.00) or SDMT (partial *ƞ*^2^ = 0.018), Table 3).

**Table 3:** Neuropsychological test scores before and after treatment with SOPT by group.

Variable	Baseline		Follow-up		*F*
	Treatment group *N* = 22	Control group *N* = 24	Treatment group *N* = 22	Control group *N* = 24	
SDMT score	37.2 (14.1)	37.8 (15.6)	36.9 (14.5)	35.9 (14.5)	*F* (1,46) = 0.8, *p = 0*.377
WAIS-IV symbol search	19.7 (8.16)	20.0 (9.66)	20.2 (8.49)	20.6 (9.50)	*F* (1,45) = 0.003, *p =* 0.954
WAIS-IV coding	41.9 (18.5)	42.9 (18.9)	41.9 (18.9)	45.1 (20.2)	*F* (1,44) = 1.72, *p =* 0.197
CVLT learning slope 1–4	1.29 (1.04)	1.08 (0.84)	1.47 (0.95)	1.11 (0.75)	*F* (1,46) = 1.71, *p = 0*.198

#### Impact of PS improvement on NLM

To examine far transfer of training related improvement, specifically the impact of SOPT on NLM, ANCOVA examined the CVLT slope at immediate follow-up as the dependent variable with the CVLT baseline as the covariate, and treatment group as the between subjects’ factor. No significant effect was noted on NLM with a small to medium effect size (partial *ƞ*^2^ = 0.037).

### Long term effects

No significant main effects or interactions were noted on the RM-ANOVA of the SDMT, symbol search and coding [2 (group: TX, pCTL) × 2 (follow-up: IFU, LTFU)], all with small effect sizes.

### Booster sessions

To examine the impact of monthly booster sessions on performance over time, we conducted a 2 (treatment group only: booster *n* = 8, non-booster *n* = 11) × 2 (follow-up: IFU, LTFU) RM-ANOVA on each outcome. Across outcomes, the interaction was not significant. However, given the very small n’s in this subgroup analysis, it is important to note that effects sizes were all in the medium range, indicating some benefit to the use of booster sessions in this population [Coding partial *ƞ*^2^ = 0.09; Symbol Search partial *ƞ*^2^ = 0.046; SDMT partial *ƞ*^2^ = 0.056; CVLT partial *ƞ*^2^ = 0.055].

## Discussion

Results of the current study showed significant improvement from pre-to post-treatment with SOPT on all subtests of the UFOV, a task similar to the training task. However, no change was evident on tests assessing near transfer to neuropsychological tests of PS and far transfer to tests of NLM. NPE 6-months post-treatment also showed no change in PS ability over time. Monthly booster sessions did not exert an impact on long-term benefit in a small subset of the sample.

As observed in the ACTIVE study (32), improved performance on the UFOV was observed in our moderate to severe TBI sample following SOPT. SOPT thus improved performance specifically on a test of PS, similar to the training task, in individuals with TBI, similar to that which has been observed in aging (32), HIV (41), and Multiple Sclerosis (27). However, it is important to note that the control group did not complete the UFOV; we thus cannot evaluate the role of practice in the noted improvement. We were unable to document transfer of these improved PS skills to neuropsychological tests of PS or further transfer to NLM. The majority of studies examining SOPT in neurological populations do not examine efficacy at the level of neuropsychological functioning (32, 42); rather existing studies, all in aging populations, focus on basic measures of PS such as the UFOV (32, 42). The few studies that do administer pre-and post-treatment neuropsychological testing have not found treatment benefits on tests of PS, including work in both aging (25) and HIV (41). The only other study to our knowledge that documented efficacy of SOPT on neuropsychological tests of PS was our work in MS, which noted a treatment effect on one measure of PS and a trend on a 2nd.

It is important to consider methodological differences between SOPT studies. Specifically, the study of SOPT in MS utilized a simple neuropsychological test of PS to evaluate near transfer, Pattern Comparison, documenting near transfer (27). The current study however, as well as the aging study that utilized neuropsychological tests (25), applied more complex measures of PS involving a larger visual array that required lateral stimulus tracking. Results on these more complex measures of PS (e.g., SDMT) were consistent with that which was observed in MS. The HIV study that utilized neuropsychological testing did not assess PS at all, but rather tested executive functioning, not noting an effect (41). Thus, in the MS study (27), in which near transfer was noted, trained tasks and near transfer neuropsychological tasks of PS were more similar to the training task than in all other SOPT studies. In fact, a treatment effect was not documented on more complex measures of PS utilized in the MS study either (e.g., SDMT) (27). There thus does appear to be consistency in the impact of SOPT on tasks similar in nature to the treatment task, in addition to far transfer to everyday life skills. Tests of everyday life functioning from the current study are beyond the scope of this paper and will be presented elsewhere. However, SOPT shows little impact on neuropsychological assessment, the traditional means of assessing change following cognitive rehabilitation.

A second consideration in interpreting the pattern of results across studies lies in the differences in cognition between TBI and both MS and healthy aging. In MS and healthy aging, PS has been identified as the primary source of cognitive change, with other cognitive deficits occurring subsequent to PS, particularly in MS samples. In TBI however, a much more complex constellation of deficits has been identified (3). Specifically in this sample of individuals with moderate to severe TBI, deficits are evidenced not only in PS, but also in mental flexibility, inhibition, working memory and episodic memory (see Table 2). This is not the case in MS and normal aging, both of which show substantially more mild deficits in other cognitive domains. It is thus possible that the severity of cognitive impairment in other cognitive domains in the current sample precluded the ability of participants to show transfer of benefit from SOPT.

In providing cognitive rehabilitation to the TBI population specifically, one treatment factor that may be important to achieving an impact on other cognitive skills and daily life is the treatment comprehensiveness. Given that most neuropsychological tasks as well as everyday life tasks, typically require multiple cognitive abilities to be completed successfully, exclusively focusing on PS, as we did in the current study, is likely not the most efficient choice of treatment to maximize impact on cognition and daily life, particularly in populations with more severe cognitive impairment. Most treatment paradigms developed to date to address PS difficulties have in fact included PS treatment, as well as addressing other cognitive domains [e.g. (43, 44),]. Study outcome measures similarly capture change in various realms of cognitive functioning, rather than focusing solely on PS (43, 44). In fact, the majority of SOPT studies do not include neuropsychological tests of PS at all (41); the degree of transfer of SOPT treatment effect to neuropsychological PS tasks is thus unknown. One could hypothesize that the lack of transfer to NLM in the current study was due to the focus of the intervention solely on PS. As discussed previously in relation to the SOPT in clinical populations (28), embedding a specific treatment, such as SOPT, within a more comprehensive treatment program potentially including metacognitive strategies has proven effective for improving far transfer and generalization in previous work with children (45), healthy older adults (46), schizophrenia (47), and cancer (48). This is indeed the approach supported by expert task forces including INCOG (24) and CRTF (23), who advocate the use of metacognitive strategies focused on daily life activities. Along with Cicerone et al. (23), we would argue that the inclusion of both metacognitive strategies along with direct training known to impact PS specifically, such as SOPT, would maximize overall benefit to the individual.

The examination of the long-term impact of monthly booster sessions post-treatment was interesting. While monthly booster sessions did not exert a significant impact on the long-term benefit of SOPT, effect sizes for the booster session comparison were medium. Importantly, delayed benefit from SOPT has been demonstrated in previous research. That is, aging work has shown that completion of booster sessions increased PS performance by 2.5 standard deviations (32), while MS work has shown delayed benefit from treatment with SOPT, documented in those initially classified as non-responders (27). Although such patterns of extended and delayed benefit were not significant in the current study, the sample sizes were extremely small, and effect sizes indicate that booster sessions show some benefit for this population. This is an important question for future research.

There were some methodological limitations in the current study that are important to mention. First, the UFOV was administered to the treatment group only and at baseline and immediate follow-up only. Administration of the UFOV at the long-term follow-up as well as administering the UFOV to all participants would have enhanced our ability to fully evaluate SOPT in this population. Second, despite efforts to obtain a larger sample, the number of participants in the current study was small, particularly when analyzing subgroups (e.g., booster sessions); a larger initial sample size would have been beneficial. Finally, there were large differences in time post injury in the current sample and time since injury was beyond what would be ideal for an intervention study in many participants.

## Conclusion

In conclusion, the current study demonstrated that training with SOPT improved task performance on a task similar to the training task (UFOV), but benefits did not extend to improvement neuropsychological tests of processing speed. Future work should seek to maximize such transfer.

## Data availability statement

The raw data supporting the conclusions of this article will be made available by the authors, without undue reservation.

## Ethics statement

The studies involving humans were approved by Kessler Foundation Institutional Review Board. The studies were conducted in accordance with the local legislation and institutional requirements. The participants provided their written informed consent to participate in this study.

## Author contributions

NC: Conceptualization, Data curation, Formal analysis, Funding acquisition, Investigation, Methodology, Project administration, Resources, Supervision, Validation, Visualization, Writing – original draft, Writing – review & editing. SC: Data curation, Formal analysis, Writing – original draft, Writing – review & editing, Supervision. CA: Data curation, Writing – original draft, Writing – review & editing, Formal analysis. KC: Data curation, Writing – review & editing, Writing – original draft, Formal analysis. SW: Data curation, Writing – review & editing, Writing – original draft, Formal analysis. NM: Conceptualization, Data curation, Project administration, Resources, Supervision, Validation, Writing – review & editing, Writing – original draft. JD: Conceptualization, Funding acquisition, Methodology, Resources, Writing – review & editing, Writing – original draft.
